# A comprehensive analysis of coding and non-coding transcriptomic changes in cutaneous squamous cell carcinoma

**DOI:** 10.1038/s41598-020-59660-6

**Published:** 2020-02-27

**Authors:** Kunal Das Mahapatra, Lorenzo Pasquali, Jonas Nørskov Søndergaard, Jan Lapins, István Balazs Nemeth, Eszter Baltás, Lajos Kemény, Bernhard Homey, Liviu-Ionut Moldovan, Jørgen Kjems, Claudia Kutter, Enikö Sonkoly, Lasse Sommer Kristensen, Andor Pivarcsi

**Affiliations:** 1 Dermatology and Venereology Division, Department of Medicine Solna, Karolinska Institutet, Stockholm, Sweden; 2 Center for Molecular Medicine, Karolinska Institutet, Stockholm, Sweden; 3 Department of Microbiology, Tumor and Cell Biology, Karolinska Institute, Science for Life Laboratory, SE-171 77 Stockholm, Sweden; 4 Unit of Dermatology, Karolinska University Hospital, SE-17176 Stockholm, Sweden; 5 Department of Dermatology and Allergology, Faculty of Medicine, University of Szeged, Szeged, Hungary; 6 Department of Dermatology, University Hospital Düsseldorf, Düsseldorf, Germany; 7 Interdisciplinary Nanoscience Center (iNANO), Aarhus University, DK-8000 Aarhus, Denmark; 8 Department of Molecular Biology and Genetics, Aarhus University, Aarhus, Denmark; 9 Research Institute of Translational Biomedicine, University of Szeged, Szeged, Hungary

**Keywords:** Squamous cell carcinoma, Long non-coding RNAs, Transcriptomics

## Abstract

Cutaneous Squamous Cell Carcinoma (cSCC) is the most common and fastest-increasing cancer with metastatic potential. Long non-coding RNAs (lncRNAs) and circular RNAs (circRNAs) are novel regulators of gene expression. To identify mRNAs, lncRNAs and circRNAs, which can be involved in cSCC, RNA-seq was performed on nine cSCCs and seven healthy skin samples. Representative transcripts were validated by NanoString nCounter assays using an extended cohort, which also included samples from pre-cancerous skin lesions (actinic keratosis). 5,352 protein-coding genes, 908 lncRNAs and 55 circular RNAs were identified to be differentially expressed in cSCC. Targets of 519 transcription factors were enriched among differentially expressed genes, 105 of which displayed altered level in cSCCs, including fundamental regulators of skin development (MYC, RELA, ETS1, TP63). Pathways related to cell cycle, apoptosis, inflammation and epidermal differentiation were enriched. In addition to known oncogenic lncRNAs (PVT1, LUCAT1, CASC9), a set of skin-specific lncRNAs were were identified to be dysregulated. A global downregulation of circRNAs was observed in cSCC, and novel skin-enriched circRNAs, circ_IFFO2 and circ_POF1B, were identified and validated. In conclusion, a reference set of coding and non-coding transcripts were identified in cSCC, which may become potential therapeutic targets or biomarkers.

## Introduction

Cutaneous squamous cell carcinoma (cSCC) is one of the most common human malignancies worldwide, with an yearly 700,000 diagnosed cases in the US alone^1^. This keratinocyte-derived cancer develops mostly on sun-exposed skin, proceeds as a progressively invasive malignancy, starting from precancerous lesions, actinic keratosis (AK), which can progress into invasive cSCC^2^. The incidence of cSCC is increasing at an alarming rate worldwide majorly due to life-style changes, an ageing population and an increase in organ transplantations, which is a major risk factor for aggressive and multiple cSCCs^3^. Although early stage cSCC is curable by surgical excision, metastatic cSCC has a poor long-term survival rate of 10–20% due to inefficacy of systemic chemotherapy^4^. Thus, there is an urgent need to identify new druggable targets and pathways in cSCC.

The most important risk factor for cSCC is a cumulative lifetime exposure to UV-B radiation. Consequently, cSCC has an extremely high mutational burden with approximately 50 mutations per mega-base coding sequence and driver mutations in key tumor suppressor genes (*TP53* and *NOTCH1/2*) and oncogenes (*HRAS* and *KRAS*)^5^. These early mutational events prime for oncogenic transformation through altered cell cycle, decreased apoptosis, all cumulatively leading towards uncontrolled proliferation of keratinocytes. Subsequent genomic alterations in oncogenes such as *EGFR and MYC* and tumor suppressors such as *TP63* and *NOTCH3* further contribute to the disease progression^5,6^. Previous transcriptome analyses have revealed thousands of protein-coding transcripts with altered expression in cSCC, but much less is known about the alterations in other types of RNAs^7,8^.

Long non-coding RNAs (lncRNAs) are a functionally diverse group of regulatory RNAs with transcript length of 200 nucleotides or longer^9^. The expression of lncRNAs is often stringently regulated in spatio-temporal manner during development^10^. Recent studies have convincingly shown vital roles for several lncRNAs not only in tissue homeostasis but also in tumor initiation, growth and metastasis^11^.

Circular RNAs (circRNAs) have recently been implicated in the regulation of gene networks with tissue-specific expression patterns^12^. CircRNAs are formed by a head-to-tail splicing event joining a 5′ splice site to an upstream 3′ splice site^13^. These molecules are exceptionally stable due to the lack of free ends and their functions are likely to be related to this structural feature. Any circRNAs have been shown to regulate gene expression in cancer via various modes of action such as decoys to sponge miRNAs and as regulators of transcription and alternative splicing^14^.

The goal of our study was to identify a reliable set of differentially expressed transcripts, including mRNAs, lncRNAs and circRNAs, in cSCC. To this end, we performed a RNA-seq analysis of cSCC and healthy skin at an unprecedented depth. Our analysis identified a large number of differentially expressed transcripts that included mRNAs, lncRNAs and circRNAs with previously uncharacterized roles in cSCC.

## Results

### Whole transcriptome profiling by RNA sequencing in cSCC and healthy skin

In order to identify alterations in the expression of protein-coding as well as non-coding genes in cSCC, RNA sequencing of cSCCs (n = 9) and unmatched healthy skin samples (n = 7) was performed using the NextSeq500-platform, generating 800 million total reads (Supplementary Table S1), which to our knowledge represents the deepest transcriptomic analysis of cSCC to date. On average 49.8 million 100 base pair (bp) paired-end reads were obtained from each sample and genome mapping was on average 55% for all samples. We performed the subsequent analysis of coding sequences (mRNAs), non-coding transcripts (lncRNAs) and circular RNAs (circRNAs) separately.

### Altered expression of protein-coding genes in cSCC

**Figure 1:**
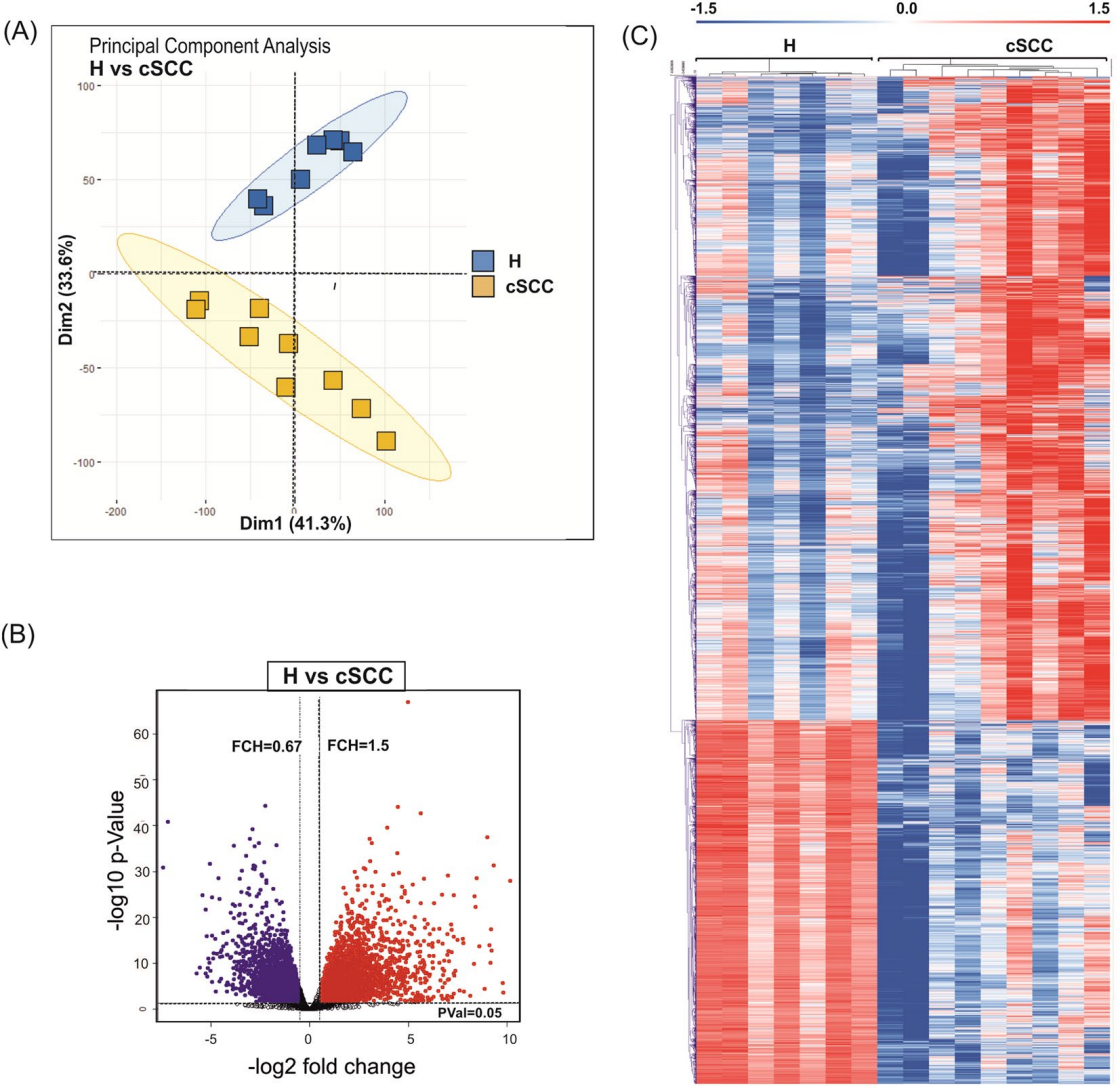
Analysis of the protein-coding transcriptome in cSCC. (**A)** Principal component analysis of samples obtained frmo healthy skin samples (H; blue) and cSCC (cSCC; yellow) based on RNA-seq data. **(B)** Volcano plot shows the result of EdgeR-analysis of all detected mRNAs (log_2_ fold change versus log_10_ nominal P-value for all detected genes). Vertical lines denote the fold change cutoff, while the horizontal line denotes the P-value cutoff. Red color represents upregulated and blue color represents downregulated coding transcripts. **(C)** Heatmap and hierarchical clustering of all differentially expressed protein-coding genes in cSCC (FDR < 0.05 and FCH > 1.5).

Principal component analysis (PCA) of all detected genes clearly separated cSCC from healthy skin samples (Fig. 1A). More variation was observed among cSCC samples as compared to samples obtained from healthy skin (H), potentially arising from an inherent heterogeneity of the disease caused by its exceptionally high mutational burden. Differential expression analysis identified 5,352 differentially expressed genes (DEGs) of which 3,419 were upregulated and 1,933 were downregulated in cSCC (linear fold-change (FCH) > 1.5, false discovery rate (FDR) < 0.05) (Fig. 1B, Supplementary Table S2). Unsupervised hierarchical clustering of protein-coding genes separated the healthy skin and cSCC samples (Fig. 1C). The DEGs included several well-known genes related to skin carcinogenesis with roles in cell motility (e.g. SNAI2, TGFBR1), extracellular matrix remodeling (e.g. BMP, MMP10), cell proliferation (e.g. MKI67, PCNA), apoptosis (e.g. BCL2, DDR1), epidermal differentiation (e.g. LCE2D, KRT10, MAF), stemness (e.g. ITGA6 and ITGB1) and inflammation (e.g. IFNGR1, IL-8/CXCL8) (Supplemental Fig. S1).

### Functional classification of deregulated protein-coding genes in cSCC

**Figure 2:**
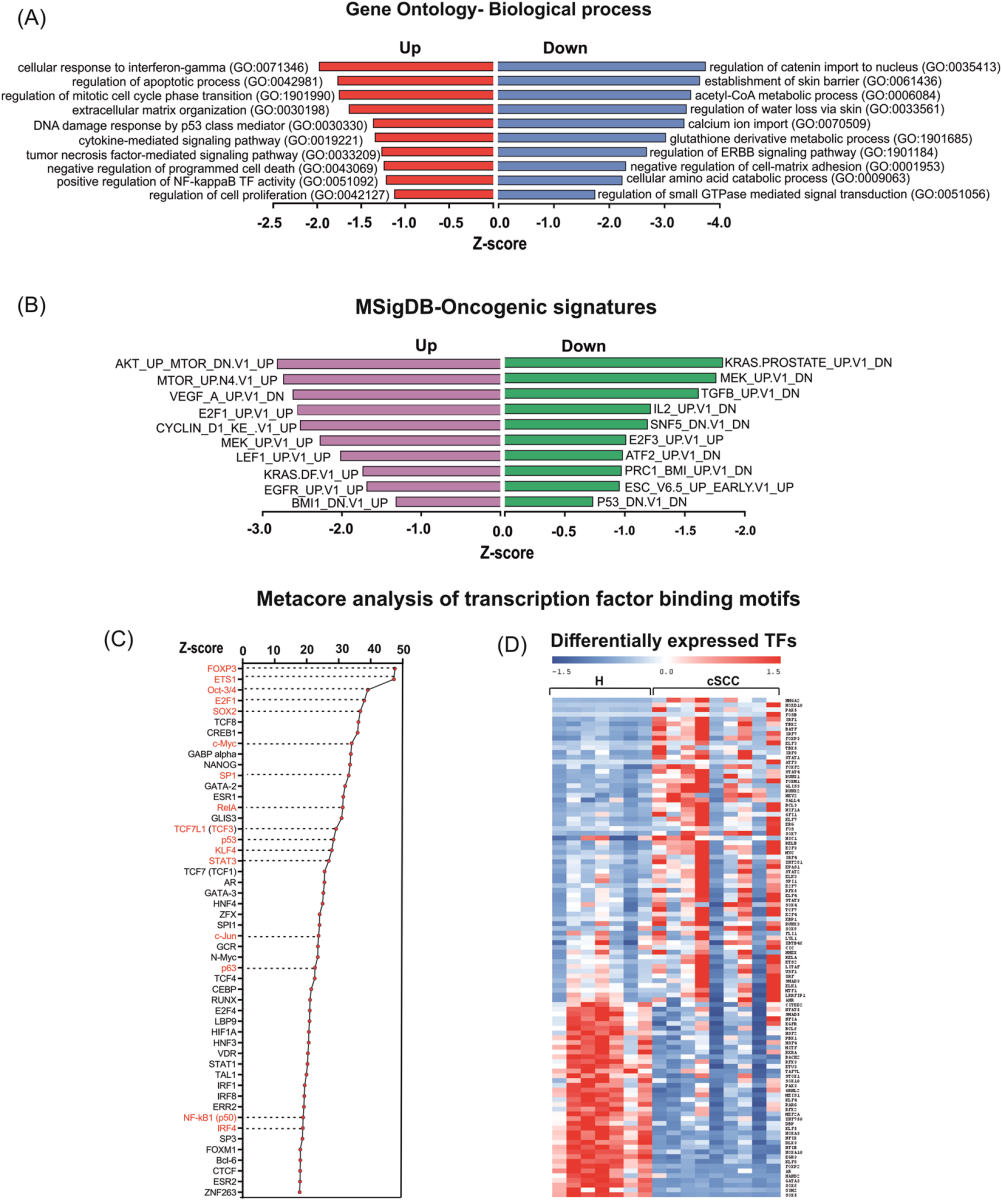
Functional classification of deregulated mRNAs and identification of differentially expressed transcription factors in cSCC. **(A)** Top 10 (*P* < 0.05) biological processes (Gene Ontology, sorted on *Z*-score) for up- and downregulated coding genes in cSCC. (**B**) Top 10 most relevant (*P* < 0.05) Molecular Signature Database (MSigDB) gene setes for up- and down-regulated coding genes in cSCC. **(C)** Top 50 transcription factors with overrepresented binding sites among differentially expressed coding genes in cSCC. Transcription factors in red color have previously been associated with cSCC pathogenesis. **(D)** Heatmap of transcription factors which are differentially expressed in cSCC (FDR < 0.05 and FCH > 1.5).

In order to get an insight into the altered biological processes in cSCC, we performed Gene Ontology (GO) enrichment analysis on the identified DEGs. Because genes with increased expression can have different regulatory and functional fucntion than downregulated genes, the gene enrichment analysis was performed on up- and downregulated genes separately (Supplementary Table S3A,B). The top enriched GO terms for the upregulated DEGs were “cellular response to interferon gamma” (GO:0071346, *P* < 0.0001), “extracellular matrix organization” (GO:0030196, *P* < 0.0001), “negative regulation of programmed cell death” (GO:0043169, *P* < 0.0001), “positive regulation of NF-kappaB TF activity” (GO:0051092, *P* < 0.0001) and “DNA damage response by p53 class mediator” (GO:0030330, *P* < 0.0001) (Fig. 2A). Interestingly, a distinct signature for interferon-response was observed among DEGs with increased expression of interferon gamma receptors (IFN-YR1/IFNGR1 and IFN-YR2/IFNGR2), downstream effector STAT1 and a number of CC (CCL2, CCL4, CCL5, CCL8) and CXC chemokines (CXCL1, CXCL10 and CXCL11) (Supplementary Fig. S1). For the down-regulated DEGs, “establishment of skin barrier” (GO:0061436, *P* = 0.003) and “regulation of water loss via skin” (GO:0033561, *P* = 0.004) were among the top enriched GO Biological processes along with processes such as “acetyl-CoA metabolic process” (GO:0006084, *P* = 0.001) and “positive regulation of canonical *Wnt* signaling pathway” (GO:0035413, *P* < 0.05).

Analysis of differentially expressed genes for enriched oncogenic signatures from the Molecular Signature Data Base revealed a strong enrichment of EGFR, KRAS, mTOR, MEK and TP53-gene signatures (Fig. 2B, Supplementary Table S3C,D). This observation supports the prevailing understanding of the molecular pathogenesis of cSCC according to which inactivation of TP53 and activation of EGFR/MAPK-signaling pathways are frequent events in cSCC that lead to activation of RAS-RAF-MEK signaling pathway^5^. Interestingly, we also observed an enrichment of SNF5-regulated genes among the DEGs. SNF5 has been previously shown to act as a part of the chromatin modifier- SWI/SNF complex to regulate cell survival in a context of p53-deficit^15^.

### Identification of differentially expressed transcriptional regulators in cSCC and validation of protein-coding transcripts

We next aimed to identify the putative upstream regulators, which could be responsible for the observed transcriptomic changes in cSCC. To this end, we performed an enrichment analysis of transcription factor (TF) binding sites among the differentially expressed genes using MetaCore. This motif-based analysis identified 519 TFs whose target genes were significantly enriched among the DEGs (*P* < 0.05, Supplementary Table S4A) out of which 105 TFs were deregulated (64 up- and 41 downregulated) at the transcript level in our RNA-seq data (Fig. 2C,D) potentially explaining a part of the observed transcriptomic changes (Supplementary Table S4B). One of the most overrepresented TF was ETS1, which has been previously shown to block terminal differentiation in keratinocytes along with induction of matrix metalloproteases^16^. Target genes of TP63, a master regulator of epidermal development and an important player in stemness and skin tumorigenesis, were also found to be significantly enriched^17^. In addition to TP63, overrepresentation of KLF4-target genes highlighted a impaired epidermal-differentiation program, which is often a primary event in cSCC^18^. TP53, the most frequently mutated gene in cSCC was also identified^19^. Several TFs with known (e.g. E2F1, c-MYC and SP1) and currently unreported functions (e.g. FOXP3, IRF1, USF1 and FOXM1) in cSCC were identified^20–22^. Enrichment of STATs (STAT3 and STAT5A) and IRF1-regulated genes can possibly contribute to the strong type I interferon response signature in our dataset. RELA was another over-represented TF, which highlighted an activation of NF-kappaB signaling in cSCC (Fig. 2A, Supplementary Fig. S1)^23^.

**Figure 3:**
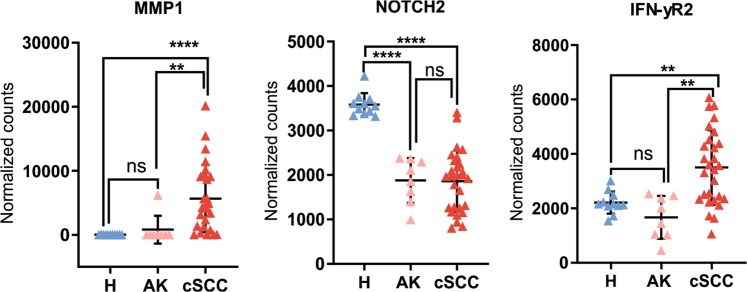
Validation of selected differentially expressed protein-coding genes. MMP1, NOTCH2 and IFN-YR2 in healthy skin (H, n = 11), AK (n = 8) and cSCC (n = 28) by NanoString nCounter assay. Target gene expression is presented as background-corrected and normalized count (threshold count of 35). *P < 0.05, **P < 0.01, ***P < 0.001, ****P < 0.0001, Mann-Whitney *U* test.

Next, to validate the results of the RNA-seq analysis, we performed NanoString nCounter assays for three DEGs on a larger cohort consisting of healthy skin (H, n =11), cSCCs, (n = 28) and AKs (n = 8) (Fig. 3, Supplementary Table S5A). These three genes were representatives for enriched biological processes such as invasiveness (MMP1), elevated immune response (IFN-YR2), and altered epidermal differentiation (NOTCH2). NanoString analysis confirmed the significant upregulation of MMP1 (undetected in healthy skin) and IFN-YR2 (1.6-fold, *P* < 0.01) in cSCC with no detectable alteretion in precancerous skin lesions (AK), whereas NOTCH2 was found to be downregulated not only in cSCC (1.8-fold, *P* < 0.0001) but even in pre-cancerous skin lesions (AK; 1.79-fold, *P* < 0.0001).

### Analysis of the long non-coding RNA landscape reveals differential expression of oncogenic and skin-specific lncRNAs

**Figure 4:**
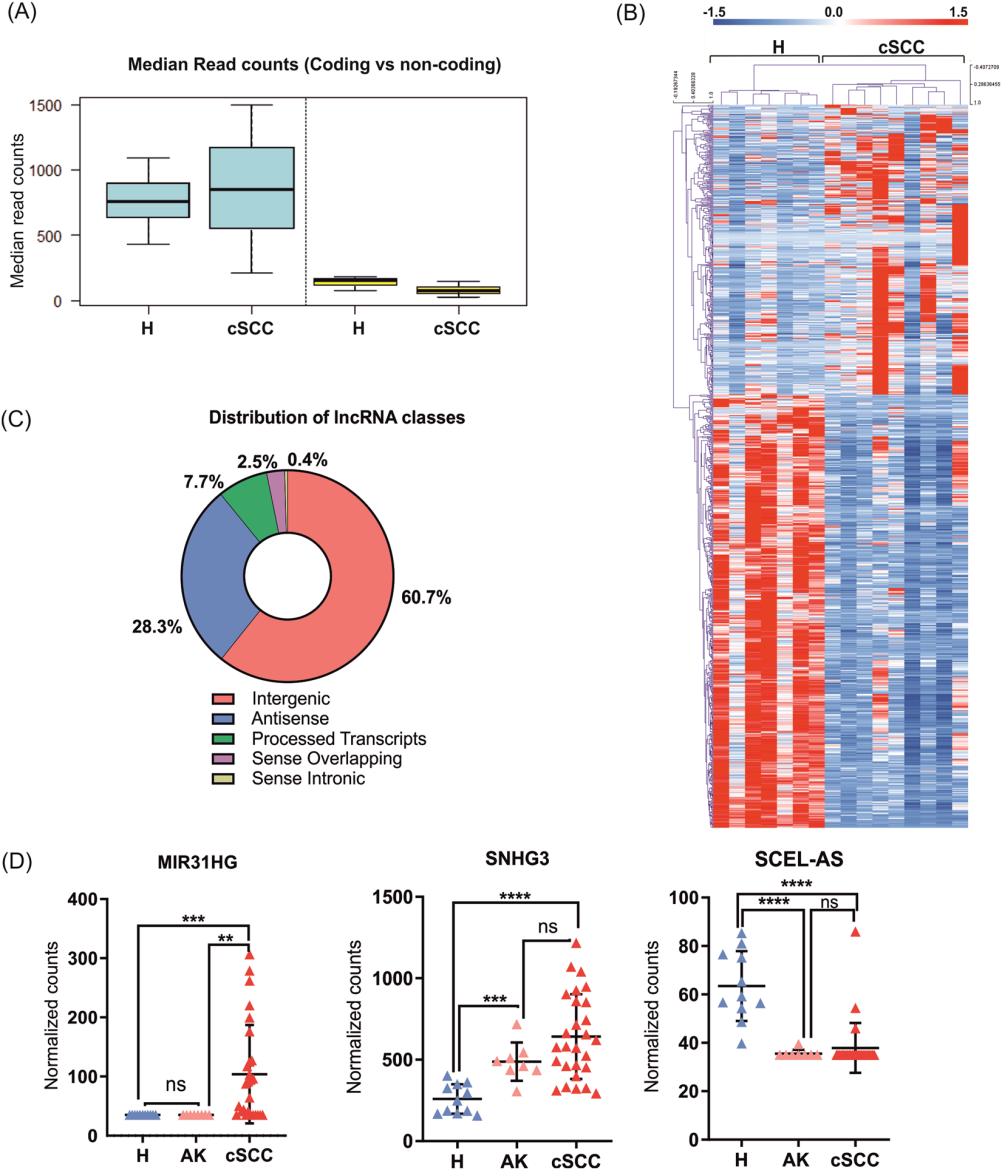
Analysis of differentially expressed long non-coding RNAs in cSCC. (**A)** Distribution of median read counts between coding and non-coding genes in healthy skin and cSCC. **(B)** Heatmap and hierarchical clustering of all differentially expressed lncRNAs in cSCC (FDR < 0.05 and FCH > 1.5). Red color represents upregulated and blue color represents downregulated lncRNAs. **(C)** Pie-chart depicting the proportion of different classes of lncRNAs with altered expression in cSCC. **(D)** Validation of selected differentially expressed lncRNAs (MIR31HG, SNGH3 and SCEL-AS) in healthy skin (H, n = 11), AK (n = 8) and cSCC (n = 28) by NanoString nCounter assay. Target gene expression is presented as background-corrected and normalized count (threshold count of 35). *P < 0.05, **P < 0.01, ***P < 0.001, ****P < 0.0001, Mann-Whitney *U* test.

Most of the previous transcriptomic studies in cSCC have only assessed protein-coding transcripts and little is known about the involvement of lncRNAs in cSCC. RNA-seq revealed a generally lower transcript abundance for lncRNAs compared to protein-coding transcripts both in healthy skin and in cSCC: while the median FPKM_mRNAs_ was 757 and 847 in healthy skin and cSCC respectively, median FPKM_lncRNAs_ was 153 and 75 (*P = *0.01 and 0.003 for H and cSCC, respectively) (Fig. 4A), in line with previous observations in other tissues^24^. Differential expression analysis identified 908 annotated lncRNAs with significantly altered expression in cSCC (FCH > 1.5, FDR < 0.05), of which 319 were upregulated and 589 were downregulated in cSCC (Supplementary Table S6). Similar to protein-coding genes, unsupervised hierarchical clustering of differentially expressed lncRNAs clearly separated the samples into healthy and cSCC groups (Fig. 4B). The most upregulated lncRNA in cSCC was RP11-493L12.5 (46.77-fold), while the most downregulated one was KB-1410C5.3/lnc-GRHL2 ( 0.005-fold). Of note, a number of lncRNAs with broad oncogenic (SNHG12, CASC9, LUCAT1 and PVT1) or tumor suppressor (such as TINCR) function were also identified to be differentially expressed in cSCC^25–28^.

Next, we classified differentially expressed lncRNAs into different subgroups based on their relative position to the nearby protein-coding genes: the most prominent classes of detected lncRNAs were intergenic and antisense (60.7% and 28.3%) followed by processed transcripts (7.7%), sense overlapping transcripts (2.5%) and sense intronic (0.4%) (Fig. 4C).

To get further insights into tissue-specific transcriptomic alterations, we calculated the tissue enrichment score (T) for all the differentially expressed protein-coding genes and lncRNAs using publicly available RNA-seq data for 28 tissues, including skin, from the GTEx database (Supplementary Table S7A,C)^29^. Although we obtained skin-enriched transcripts for both protein-coding genes and lncRNAs, lncRNAs displayed a higher degree of skin-specificity (Ts > 0.9) as indicated by the relative frequency of their skin enrichment scores (Supplementary Fig. S3, Table S7B,D). Interestingly, analysis of the top 50 skin-enriched mRNAs and lncRNAs revealed that most of them were downregulated in cSCC (Supplementary Figs. S4 and S5). These skin-specific transcripts (e.g. RP13-455A7.1, CHODL-AS1, RP11-73G16.2) are largely uncharacterized in cSCC and therefore require further investigation.

Next, we aimed to identify the possible regulators of the differentially expressed lncRNAs in cSCC. Motif enrichment analysis (Supplementary Fig. S5) using their upstream regulatory regions resulted in a significantly high enrichment for CREB, ATF1 and NRF1 associated motifs for upregulated lncRNAs. A previous study has shown that CREB - a transcriptional co-activator of c-Jun regulates keratinocyte proliferation and differentiation^30^. For the downregulated lncRNAs, we observed a significant motif enrichment for SP1/SP4 transcription factors which have been demonstrated to regulate cell-specific gene expression during keratinocyte differentiation^31^.

Three differentially expressed lncRNAs were validated in an expanded cohort of patient samples that also included pre-cancerous skin lesions (AK), using NanoString nCounter assays (Fig. 4D). SCEL-AS1 was chosen as a representative of skin-enriched lncRNAs. MIR31HG and SNHG3, both of which have been reported to play oncogenic role in other cancers, were 2.5- and 2.3-fold upregulated (*P* < 0.0002 and *P* < 0.0001), respectively. Although MIR31HG was not altered, SNHG3 was found to be upregulated (1.9-fold, P = 0.0003) already in the AK samples. SCEL-AS1, found to be significantly downregulated in cSCC and AK samples (1.5-fold, *P* < 0.0001) is located antisense to Sciellin (*SCEL*) with enriched expression in human skin and esophagus (https://www.ncbi.nlm.nih.gov/gene/104355296/?report = expression).

### Identification of novel circRNAs in healthy skin and cSCC

Detection and quantification of circRNAs in our RNA seq dataset was done using a stringent version of the find_circ pipeline as previously described^32^. In total, we identified 227 and 150 circRNAs supported by at least an average of five backsplicing junction-spanning reads in healthy skin and cSCCs, respectively (Supplementary Table S8A,B). Notably, most of these, 197/227 (86.8%) and 139/150 (92.7%), were also detected by the circExplorer pipeline indicating that these are high-confidence circRNAs (data not shown). Among the top 50 most abundant circRNAs in either sample type, we observed several circRNAs (e.g. CDR1as, circ_HIPK3, circ_CDYL, circ_FAT1, circ_LPAR1 and circ_SLC8A1) that are highly expressed in several tissues in RNA-seq data from the ENCODE consortium (known as the top 10 alpha circRNAs (Supplementary Fig. S6A,B)^33^. In addition to annotated circRNAs, our analysis also identified six novel circRNA candidates, which are not annotated in circBase, derived from *IFFO2*, *PLIN4*, *DMKN*, *METRNL*, *KRT1* and *POF1B* genes (Supplementary Table S8). Of note, two of the novel circRNA (circ_IFFO2 and circ_PLIN4) were relatively abundant (>1 RPM) in the normal skin.

**Figure 5:**
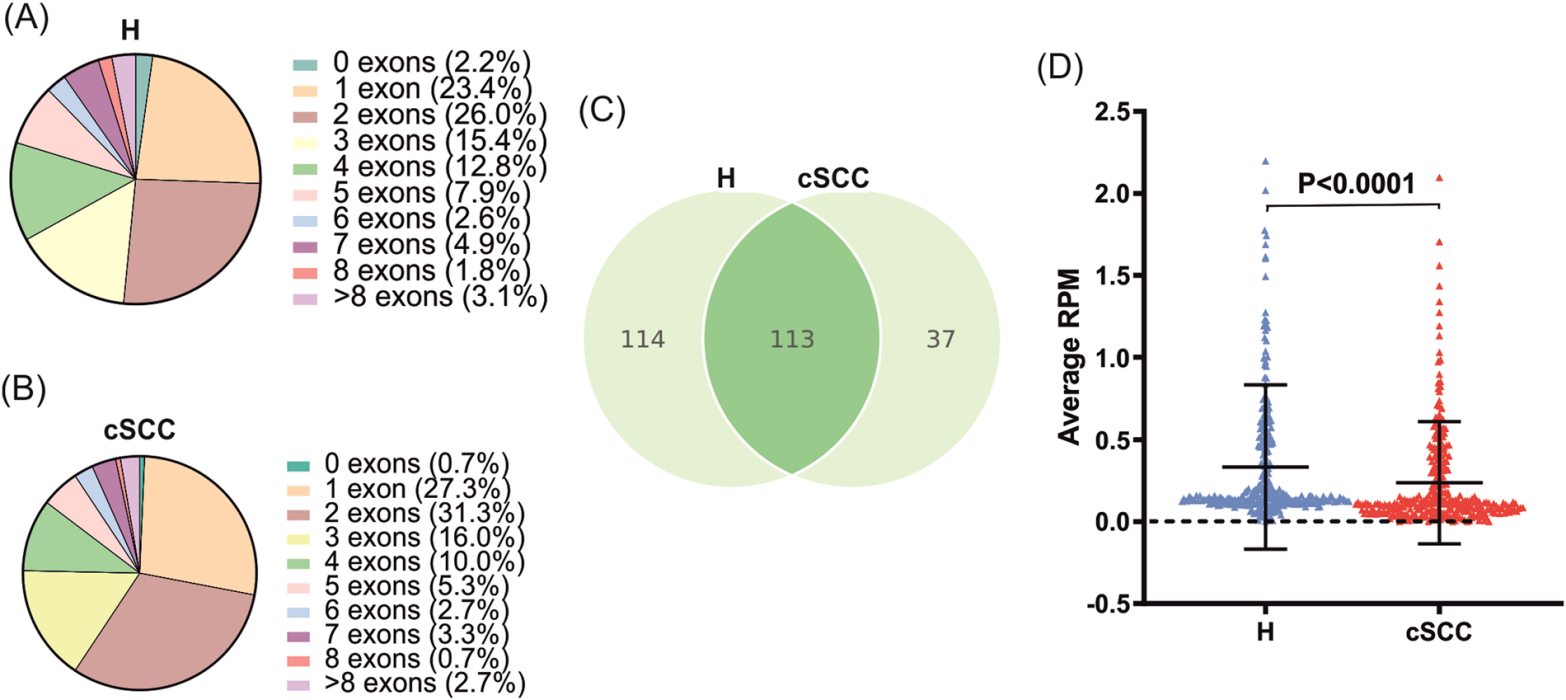
Detection of abundant circRNAs in healthy skin and cSCC. Pie charts showing the distribution of the numbers of exons annotated within the back-splicing junction of the high abundance circRNAs in healthy skin **(A)** and in cSCC **(B). (C)** Venn-diagram showing the overlap between the high abundance circRNAs detected in the normal skin biopsies (left) and the cSCC biopsies (right). **(D)** Column scatter plot showing the average RPM for the 264 unique high abundance circRNAs detected in the normal skin- and cSCC biopsies combined. Mann-Whitney *U* test.

Evaluation of exon structures of the detected circRNAs revealed that circRNAs consisting of two exons was the most frequently observed population in both healthy and cSCC samples (Fig. 5A,B), similar to previous observations in other tissues and tumor types^32^. In terms of circRNA producing-loci, find_circ identified that the 227 circRNAs in healthy skin from 210 host genes and that the 150 circRNAs in cSCC originated from 142 host genes. Of note, 37 (16.3%) and 22 (14.7%) circRNAs were on average expressed at higher levels than their linear host genes (Circular to Linear ratio (CTL) > 1) in the normal skin and cSCC, suggesting their slow turnover or increased transcriptional output.

In total, 264 high abundance circRNAs were detected in cSCC and normal skin samples combined. Although the overlap between the circRNAs detected in cSCC and in normal skin was substantial (42%), many were unique, mainly expressed in the normal skin (Fig. 5C). To get an insight into their expression pattern, we plotted the expression value of these 264 circRNAs and observed a significant reduction in cSCC samples compared to healthy skin (Fig. 5D).  This apparent downregulation in circRNA adundance prompted us to investigate whether circRNA-biogenesis could be altered in cSCC. Indeed, we observed that among the DEGs key negative regulator of circRNA-biogenesis ADAR is significantly upregulated (2.03-fold, FDR = 4x10^−8^) in cSCC while positive regulators such as MBNL (0.66-fold, FDR = 4.2x10^−5^) and ESRP1 (0.52-fold, FDR = 1.2x10^−4^) were downregulated (Supplementary Fig. S7) thereby providing a potential explanation for the large-scale decrease in circRNA abundance.

### Differentially expressed circRNAs are mostly downregulated in cSCC

**Figure 6:**
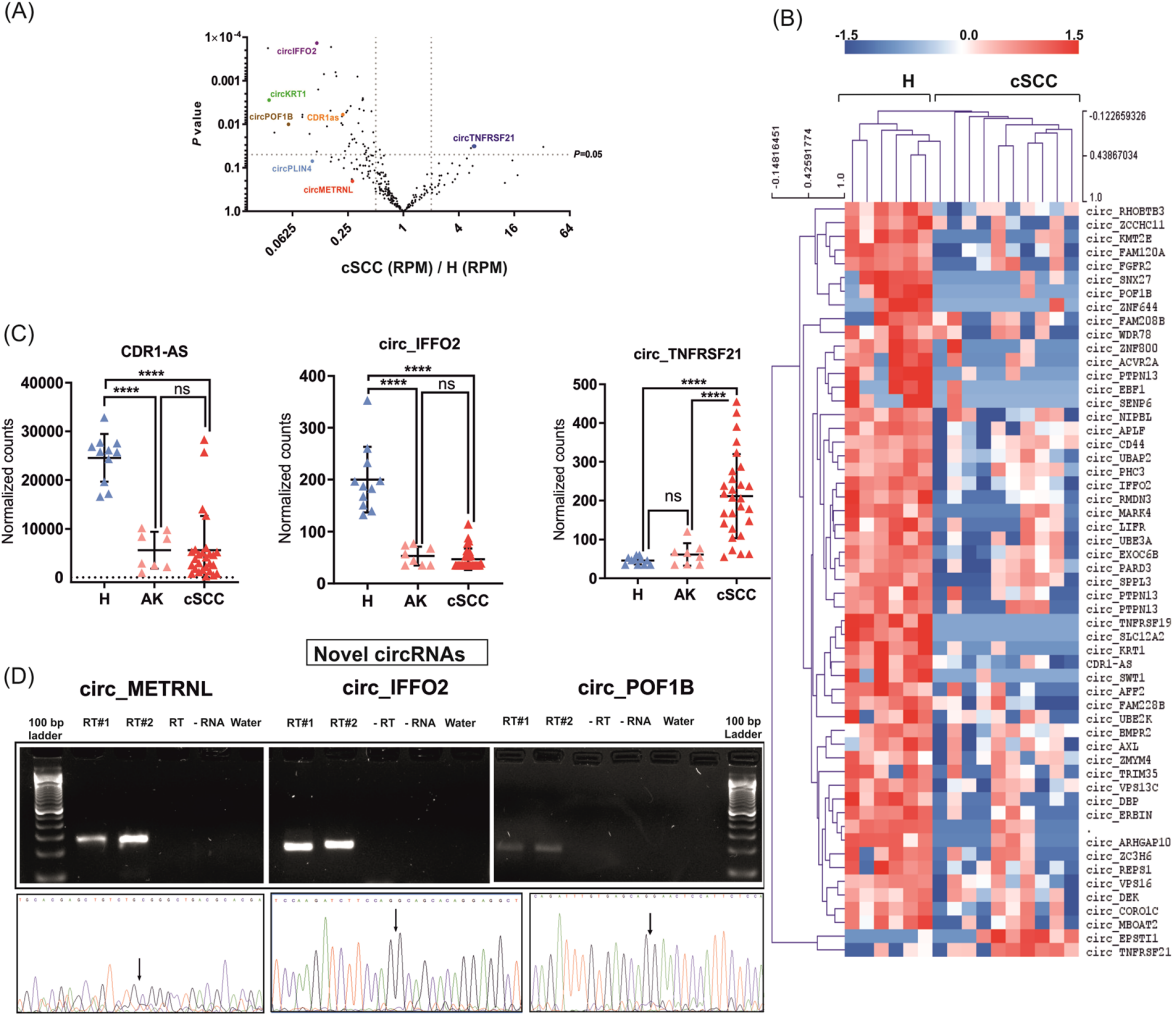
Analysis of differentially expressed circRNAs in cSCC. (**A)** Volcano plot showing the fold changes in RPM vs *P*-values for the 264 unique high abundance circRNAs with the exception of circRNAs that were not expressed in either of the sample groups. **(B)** Heatmap and hierarchical clustering of all 55 differentially expressed circRNAs in cSCC. Red color represents upregulated and blue color represents downregulated circRNAs. **(C)** Validation of selected differentially expressed circRNAs (CDR-AS1, circ_IFFO2 and circ_TNFRSF21) by NanoString nCounter assay in healthy skin (H, n = 11), AK (n = 8) and cSCC (n = 28). Target gene expression is presented as background-corrected and normalized counts (threshold count of 35). ****P < 0.0001, Mann-Whitney *U* test. **(D)** Validation of the novel circRNAs (circ_METRNL, circ_IFFO2 and circ_POF1B) detected in our analysis by PCR using divergent primers and Sanger sequencing. The agarose gel image showed the expected size of PCR product present only in the reverse-transcribed samples. Below the agarose gel images, corresponding Sanger sequencing chromatograms across the backsplicing junction are shown. Arrows indicate the back-splicing junctions. Gel images for respective circRNA transcripts were cropped from the different parts of a single gel.

Differential expression analysis identified 55 circRNAs with significantly (*P* < 0.05) altered expression in cSCC. In line with the generally lower abundance of circRNAs in cSCC (Fig. 5D) almost all differentially expressed circRNAs, 53/55 were downregulated and only two were upregulated in cSCC relative to healthy skin (Supplementary Table S8C and Fig. 6A). Hierarchical clustering of differentially expressed circRNAs clearly separated the samples into two groups, corresponding to healthy and cSCC samples (Fig. 6B). The most downregulated circRNAs included both well-characterized cancer-associated circRNAs, such as CDR1as, as well as novel circRNAs, which were derived from host genes *IFFO2*, *KRT1* and *POF1B*^34^. In terms of fold change, the most significantly downregulated circRNA was *IFFO2* ( 0.11-fold, *P* < 0.0001) while the mostupregualted was circ_EPSTI with a 33-fold increase in expression (*P* < 0.03; Fig. 6A).

To investigate whether the altered expression of circRNAs reflects the altered expression level of their host genes, fold changes in RPM were plotted against circular to linear (CTL) ratios (Supplementary Fig. S8). Comparison of linear and circular transcript produced from the same gene showed that the majority of the differentially expressed circRNAs were changed independently of their host genes.

### Validation of annotated and novel circRNAs in cSCC

To validate the findings obtained from circRNA identification and differential expression analysis, next we analyzed the expression of three differentially expressed circRNAs. The expression of CDR1as, the most well-characterized member of the circRNA family, as well as two novel circRNAs identified in this study, circ_IFFO2 and circ_TNFRSF21, in healthy skin (H, n = 11), precancerous lesions (AK, n = 8) and cSCC (n = 28) was analyzed using NanoString assays (Fig. 6C) as this assay is particularly well-suited for circRNA quantification since no enzymes are used, which can introduce amplification bias^35^. In accordance with the results of RNA-seq, CDR1as and circ_IFFO2 were found to be 6.7-fold and 5.3-fold downregulated (*P* < 0.0001), while circ_TNFRSF21 was 4.6-fold (*P* < 0.0001) upregulated in cSCC comapred to healthy skin (Fig. 6C). Interestingly, our validation data also suggested that downregulation of CDR1as and circ_IFFO2 might be an early event in cSCC progression as they were downregulated in precancerous skin lesions 4.7 and 3.7-fold downregulation in AK, respectively, P < 0.0001). Using divergent primer-based PCR amplification and Sanger sequencing, the back-spliced junction of three of the novel circRNAs detected in our analysis were confirmed (circ_IFFO2, circ_METRNL and circ_POF1B) (Fig. 6D, Supplementary Table S9, Fig. 9).

## Discussion

The molecular characterization of cSCC is an important step towards the understanding of dysregulated pathways and identifying key drivers of the disease. Although previous profiling experiments in cSCC have identified a large number of differentially expressed genes, the field has been hampered by the little consensus among these studies^7,8,36^. Here, we performed deep sequencing-based extensive analysis of transcriptomic changes in cSCC, which allowed us to identify differentially expressed protein-coding genes, including many, which were not detected in previous transcriptomic analyses, as well as a large number of lncRNAs and circular RNAs. In accordance with previous observation, we also identified a small overlap of commonly regulated differentially expressed genes in our dataset as compared to previous profiling studies (data not shown) which is possibly due to the heteregeouns nature of cSCC, along with variation in sampling and profiling methods. Interestingly, we identified a set of differentially expressed mRNAs and lncRNAs, which display skin-specific expression, suggesting function in skin development and epidermal differentiation and representing interesting targets for functional investigation.

The functional annotation of differentially expressed protein-coding genes identified enrichment of biological pathways related to altered barrier function, extracellular matrix assembly, decreased apoptosis and increased cell proliferation, indicating the breakdown of the differentiation program and associated cancer hallmarks, in line with the previous studies^5^. Interestingly, a large number of genes related to the inflammatory response and cytokine mediated signaling pathway were enriched among genes overexpressed in cSCC, including pro-angiogenic CXC chemokines such as CXCL1, CXCL10 and CXCL11 which could be involved in neo-angiogenesis^37^. Similarly, a number of CC chemokines (e.g. CCL2 and CCL5) with known oncogenic capacity were significantly upregulated^38^. Not all chemokines were overexpressed in cSCC: RNA seq results confirmed the decreased expression of CCL27 in cSCC, a chemokine earlier identified to play a role in tumor immune escape^39^.

Analysis of oncogenic signatures revealed *EGFR* and *KRAS* activation, consistently with the key role of the EGFR/MAPK pathway in epidermal carcinogenesis^5^. We observed an upregulation of the key markers of progenitor-cell related genes, e.g. ITGA6, ITGB1, CDKN1A and CDKN2A in cSCC, suggesting an expansion of progenitor-cell like population^40^. Moreover, activation of NF-kappaB signaling was also identified, mirrored by the overexpression of p65 and upregulation of several direct targets (ICAM1, NFKB2, NFKBIA, RELB, IRF1 and BIRC3) consistent with its proinflammatory and prosurvival role in cSCC^23^.

We identified a large number of transcription factors with altered expression in cSCC and thereby offer a potential mechanistic explanation for the expression changes we observed for thousands of mRNAs and lncRNAs. These differentially expressed transcription factors also represent a potential link between the genomic alterations in a relatively small number of driver genes in cSCC and the large-scale transcriptomic alterations in the disease. Many of the dysregulated transcription factors are related to skin development or skin carcinogenesis (e.g. ETS1, SP1, TP63, TP53, AP1 and TCF3)^19,22,41,42^. Similarly, several known regulators (e.g. KLF4, ZNF750, GRHL2) of the proliferation-differentiation switch in the epidermis were also altered at the transcript level. Importantly, we observed altered expression of key regulators of pluripotency and stemness (e.g. MYC, SOX2 and OCT-3/4), potentially revealing an activation of the stem-cell like program in cSCC, a feature associated with cellular transformation^40^. Increased expression of the key components of the JAK-STAT pathway (STAT1 and STAT3) further corroborates the prominent cytokine activation signature observed in the GO analysis.

In addition to the transcription factors with established roles in the disease, several less characterized transcription factors were predicted to regulate a large number of the genes in cSCC. An example is FOXM1 that has been shown to regulate cell proliferation and senescence in healthy keratinocytes and implicated in cell invasion and metastasis in head and neck squamous cell carcinoma, a disease resembling cSCC in several aspects^43^. Another less well-characterized TF whose expression was increased in cSCC was USF1, a leucine-zipper-family TF, which has been described to regulate p53 stability^44^. However, its role in cSCC has not been investigated yet.

In terms of tissue specificity, although protein-coding genes were largely non-specific (85%, Supplementary Table S7), a number of skin-specific transcripts were also identified (0.23%). As expected, majority of them were downregulated in cSCC, with many of them functioning as key players in epidermal differentiation (LCE1D, FLG, KRT77, KRT10, ALOEX3 etc.). In addition to the expected downregulation of terminal differentiation -associated transcripts, this meta-analysis also revealed that IL-37, a negative regulator of innate immunity with hitherto unexplored function in cSCC, has a strong skin-specific expression^45^. Future studies should address their functional role or prognostic value in cSCC.

Although many lncRNAs display highly lineage and cell type-specific expression pattern, we observed an increased expression of several lncRNAs dysregulated in a broad range of solid tumors, such as MIR31HG, which regulates senescence, as well as CYTOR (LINC00152), PVT1, HOXA-AS2 and SNHG12, which can regulate various cancer hallmarks^25,28,46,47^. Moreover several lncRNAs, reported to be upregulated in the primary and metastatic cSCC cell lines compared to progenitor keratinocytes^48^ (e.g. LINC00346, PVT1, SNHG12, ZFAS1 and TUG1) were significantly upregulated in cSCC, suggesting that some of the disease-related molecular alterations are comparable between cell lines and primary cells. Comparison of the differentially expressed lncRNAs with a list of altered lncRNAs in cSCC from a previous microarray-based study with a smaller sample size (n = 3), revealed a set of 51 upregulated (e.g. PVT1, HIF1A-AS2, HOXD-AS2, CYTOR) and 73 downregulated lncRNAs (e.g. SNRK-AS1, BDNF-AS, GUSBP11) consistently detected across the two studies^49^, representing a common set of cSCC-associated lncRNAs.

We identified a set of skin-enriched lncRNAs (e.g. RP13-455A7.1, RP11-73G16.2, CHODL-AS) with dysregulated expression in cSCCs. Since tissue-restricted lncRNAs often play crucial homeostatic function, the identified skin-enriched lncRNAs with altered expression in cSCCs, may also have important function in skin development and homeostasis. In this list, we also observed the presence of TINCR – a lncRNA which is required for proper epidermal differentiation^50^.

Our circRNA analysis detected 227 and 150 high confidence circRNAs in healthy skin and cSCCs, respectively. In addition to the near-ubiquitously expressed circRNAs such as CDR1as and circSMARCA5, we observed several circRNAs (circ_PARD3, circ_APLF and circ_MAP3K1) with high expression in the skin^51,52^. Apart from annotated circRNAs, we identified novel candidates derived from *IFFO2, METRNL, KRT1* and *POF1B* genes, which with the exception of circ_KRT1, we validated. Two of the host genes of these novel circRNAs- *POF1B* and *IFFO2*, are highly expressed in skin and esophagus tissues with stratified epithelia. Therefore, it would be interesting to study whether the circular counterparts also have such tissue-restricted expression and function.

We observed a general reduction of circRNA abundance in cSCC compared to normal human skin. One possibility for the overall decrease in circRNAs can be the dysregulation of their biogenesis. Indeed, we found that ADAR, a double-stranded RNA-binding protein that has been previously shown to suppress back-splicing events, was upregulated in the cSCC samples^53^. Involvement of pre-mRNA processing machinery led us to perform an intersection analysis with known splicing regulators where we found downregulation of ESRP1 and MBNL1 - two previously reported positive regulators of circRNA biogenesis^54^. Alternatively, it is also possible that the observed downregulation of circRNAs is caused by their high stability and inefficient biogenesis preventing them from reaching steady-state levels in the highly proliferative cancer cells^55^.

One of the most significantly downregulated circRNAs was CDR1as which has been previously shown to act by inhibiting tumor suppressor miR-7^51^ and also can be negatively regulated by the same miRNA cellular context^56^. Since CDR1as has been previously shown to modulate EGFR and KLF4 in breast cancer development, and these genes play importat roles also in the skin, it will be interesting to assess its potential tumor suppressive effect in the context of cSCC. One of the most differentially expressed circRNAs- circ_EPSTI1 was highly overexpressed (32-fold) in cSCC. Its host gene (*EPSTI1*) is an IFN-response gene which has been shown to promote tumor invasion and metastasis in various cancer types^57^. It is tempting to investigate whether the circRNA also have a function to synergize or antagonize with the host gene. Upregulation of circ_TNFRSF21 seems to be co-regulated with theexpression of its host gene in cSCC. In addition to CDR1as, two other downregulated circRNAs, circ_UBAP2 and circ_SNX27 have been previously demonstrated to have oncogenic effects in other cancer types^58,59^.

Our analysis showed that a number of circRNAs related to epidermal differentiation (circ_MBOAT2, circ_PTPN13 and circ_ACVR2A) were decreased in cSCC (32). Since altered epidermal differentiation program is an early event during the development and progression of cSCC, these circRNAs are promising candidates for functional characterization^32^. Comparison of results from our circRNA-analysis with that of a hybridization array-based circRNA profiling study with smaller sample size (n = 3) in cSCC revealed only one common deregulated circRNA (circ_ACVR2)^60^. This might be due to our stringent filtering criteria (more than five backsplicing junctions), large difference in the sample number and the different detection methods, as array-based circRNA profiling does not have the potential to identify novel circRNAs.

In summary, our study provides a comprehensive dataset of deregulated protein-coding genes, along with linear and circular non-coding RNAs in cSCC. The findings entail the need for a future investigation to dissect the changes in lncRNAs and circRNA landscape during various stages of cSCC progression including precancerous lesions, cSCC *in situ* and different grades of cSCC. The present study identifies previously uncharacterized lncRNAs and circRNAs with altered expression, which could be utilized to identify novel therapeutic targets in cSCC and will contribute to a better understanding of the molecular pathogenesis of cSCC.

## Methods

### RNA isolation from patient samples and RNA integrity assessment

Four mm punch biopsies were collected from 18 healthy donors and 28 patients with primary cSCC, at the department of Dermatology, Karolinska University Hospital, Stockholm, Sweden and the department of Dermatology and Allergology, Faculty of Medicine, University of Szeged, Szeged, Hungary. All the actinic keratosis samples included in the validation cohort were collected at the department of Dermatology, University Hospital Düsseldorf. All patients and healthy volunteers provided written informed consent for sample collection. Details of the samples used for whole transcriptome sequencing can be found in Supplementary Table S1 and Supplementary Table S10 (for extended cohort). Snap frozen punch biopsies were homogenized using TissueLyser LT (Qiagen) followed by RNA extraction using the miRNeasey mini kit (Qiagen). RNA concentrations were measured using a Nanodrop One (Thermo Fisher Scientific) instrument and RNA integrity was assessed on the Bioanalyzer 2100 (Agilent Technologies, Santa Clara, CA, USA) using the Nano 6000 kit.

### Evaluation of tissue specificity

 Expression value of protein coding and non-coding transcripts were obtained from the GTEx portal. Transformed fibroblasts and lymphocytes were removed from the list, as they did not represent any single tissue. For adipose tissue, brain, heart and artery, average RPKM values were calculated as an average of the different components of the same tissue/organ. Similarly, average expression value was calculated from sun-exposed and non-exposed skin samples. The tissue specificity (*T*) of a gene in tissue was calculated as the fraction of expression (RPKM) relative to the sum of its expression in all 28 tissues.

### NanoString nCounter assay

A custom CodeSet of capture and reporter probes was designed to target a unique 100 nucleotide long exon spanning regions in mRNAs, lncRNAs and backsplicing junctions of differentially expressed circRNAs. Five reference genes (RPLP0, PUM1, SF3A1, GUSB and ALAS1) were included for normalization. Probe-set and RNA hybridization reaction were performed according to the manufacturer’s instructions; complexes were purified, immobilized on nCounter cartridges and quantified using the digital analyzer. Mean of the negative controls were subtracted while analyzing the data using nSOLVER 3.0 software (NanoString Technologies, Seattle, WA, USA). Positive control normalization was performed using the geometric mean of all positive controls with the exception of the control named F, as recommended by the manufacturer. Finally, a second normalization was performed using the geometric mean of the three linear reference genes (ALAS1, PUM1 and SF3B1) having the lowest coefficient of variance percentage (%CV).

Further details are available in Supplementary Materials Section.

### Ethics approval and consent to participate

The study was approved by the Regional Committee of Health Research Ethics (Stockholm, Sweden, Szeged, Hungary and Düsseldorf, Germany), and the study was performed in accordance with the Declaration of Helsinki. All patients and healthy volunteers provided written informed consent.

## Data availibility

All data generated and analyzed in this study are included in this article and in the corresponding Supplementary Files. Normalized read counts, raw data and relevant metadata files are deposited to NCBI’s Gene expression Omnibus (Accession number: GSE139505).

## Supplementary information

Supplementary Information.

S1.

S2.

S3A.

S3B.

S3C.

S3D.

S4A.

S4B.

S5.

S6.

S7A.

S7B.

S7C.

S8A.

S8B.

S8C.

S9.

S10.
