# Supplementary material for: A comprehensive analysis of coding and non-coding transcriptomic changes in cutaneous squamous cell carcinoma
Source: Sci Rep. 2020 Feb 27;10:3637. doi: 10.1038/s41598-020-59660-6 (PMC7046790; doi:10.1038/s41598-020-59660-6)
Supplement: Supplementary file 1 — Supplementary Information. [file 41598_2020_59660_MOESM1_ESM.docx]

**A comprehensive analysis of coding and non-coding transcriptomic changes in cutaneous squamous cell carcinoma**

Kunal Das Mahapatra, Lorenzo Pasquali, Jonas Nørskov Søndergaard, Jan Lapins, István Balazs Nemeth, Eszter Baltás, Lajos Kemény, Bernhard Homey, Liviu-Ionut Moldovan, Jørgen Kjems, Claudia Kutter, Enikö Sonkoly, Lasse Sommer Kristensen and Andor Pivarcsi

**SUPPLEMENTARY INFORMATION**

**METHODS**

**Whole transcriptome sequencing**

The library preparation was done using Illumina TruSeq® Stranded Total RNA (with Ribo-Zero Gold) preparation kit at Exiqon Services, Denmark. 100 ng of total RNA was depleted of rRNAs using ribo-zero gold magnetic bead based capture-probe system (Illumina Inc.). The remaining RNA (including mRNAs, lincRNAs and other RNA species) was subsequently purified (RNAcleanXP, Beckman Coulter) and fragmented using enzymatic fragmentation. Then first strand synthesis and second strand synthesis were performed and the double stranded cDNA was purified (AMPure XP, Beckman Coulter). The cDNA was end repaired, 3’ adenylated and Illumina sequencing adaptors ligated onto the fragments ends, and the library was purified (AMPure XP). The stranded libraries were amplified with PCR and purified (AMPure XP). The libraries size distribution was validated and quality inspected on a Bioanalyzer. Sequencing was performed on NextSeq 500 instrument using v2 reagent kits according to the manufacturer instructions (Illumina Inc.).

RNA-seq alignment was performed by Exiqon/Qiagen using their XploreRNA pipeline based on the Tuxedo suit ^1^. Bowtie2 (v.2.2.6), and Samtools (v.1.2) were used for data filtering, read mapping and splice junction detection, while transcript assembly was done using Cufflinks suite (v2.2.1) (genome version: canonical chromosomes from GrCh37.p13). Identified linear transcripts were separated into coding and non-coding transcripts based on assigned gene biotype attributes in ENSEMBL (GrCh37, Release 95, January 2019), using BiomaRt (v2.38.0). Differential expression analysis was performed by the Bioconductor package edgeR (v3.24.3) ^2^. TMM (Trimmed Mean of M-values) normalization method was employed to account for the cDNA library size variation. For multiple hypothesis correction, Benjamin-Hochberg approach was used. All the heat-maps and hierarchical clustering were done using Multiple Experiment Viewer (http://mev.tm4.org/).

To identify circular RNAs in healthy skin and in SCC, we re-analyzed the whole transcriptome sequencing data using a stringent version of the find_circ (v1.2)^3^ and the CIRCexplorer pipeline (v1.1.10) ^4^. All circRNA data analyses were based on the Find_circ pipeline and circRNA candidates supported by at least five backsplicing junction-spanning reads on average per sample were defined as high abundance circRNAs. All circRNA candidates not detected by CIRCexplorer were manually inspected to exclude obvious mapping artifacts as previously described ^5^. Reads per million (RPM) refers to sequencing reads aligning across the particular backsplicing junction divided by the total number of reads multiplied by one million. The circular-to-linear (CTL) ratios were defined as the number of reads spanning the particular backsplicing junctions divided by the average linear reads spanning the splice donor- and splice acceptor sites of the same backsplicing junction plus one (to avoid division by zero). Comparison between the expression levels of the high abundance circRNAs in the cSCC- and healthy skin biopsies were done using a Mann Whitney test. The volcano plot was generated by one unpaired t test per circRNA individually without assuming consistent standard deviation and without correction for multiple testing. Linear regression was used to assess the potential correlation between fold changes in RPM and fold changes in CTL ratios employing an f test to investigate if the slope was significantly non-zero.

**Enrichment analysis**

Enrichemnt of biological processes (GO Biological process 2018) and MSigDB oncogenic signatures in the upregulated and downregulated genes was analysed using Enrichr ( v1.0) and Metacore (Thomson Reuters, v19.1)^6^. Enriched terms with *P*<0.05 upon Multiple-hypothesis correction were considered significant.

### Motif enrichment analysis for lncRNAs promoters

DNA sequences overlapping with lncRNA promoter regions (1000 bp upstream of the transcription start site of the lncRNAs) were acquired using BEDTools (v2.27.1)^7^. Overrepresented motifs and their enrichment was determined using Gimmeroc from the GimmeMotifs package (v0.13.1)^8^ using as background a set of 1000 random 1000bp sequences from the human genome (hg19) with similar GC content as the DE lncRNA promoters.

**PCR amplification and Sanger sequencing**

Total RNA from patient samples were transcribed using M-MLV reverse transcriptase (Thermo Fisher Scientific). The PCR product was verified in a 2% agarose gel, then cleaned up using QIAquick PCR Purification Kit (Qiagen) and sequenced in both direction (GATC Biotech, Konstanz, Germany).

**SUPPLEMENTARY FIGURES**

**
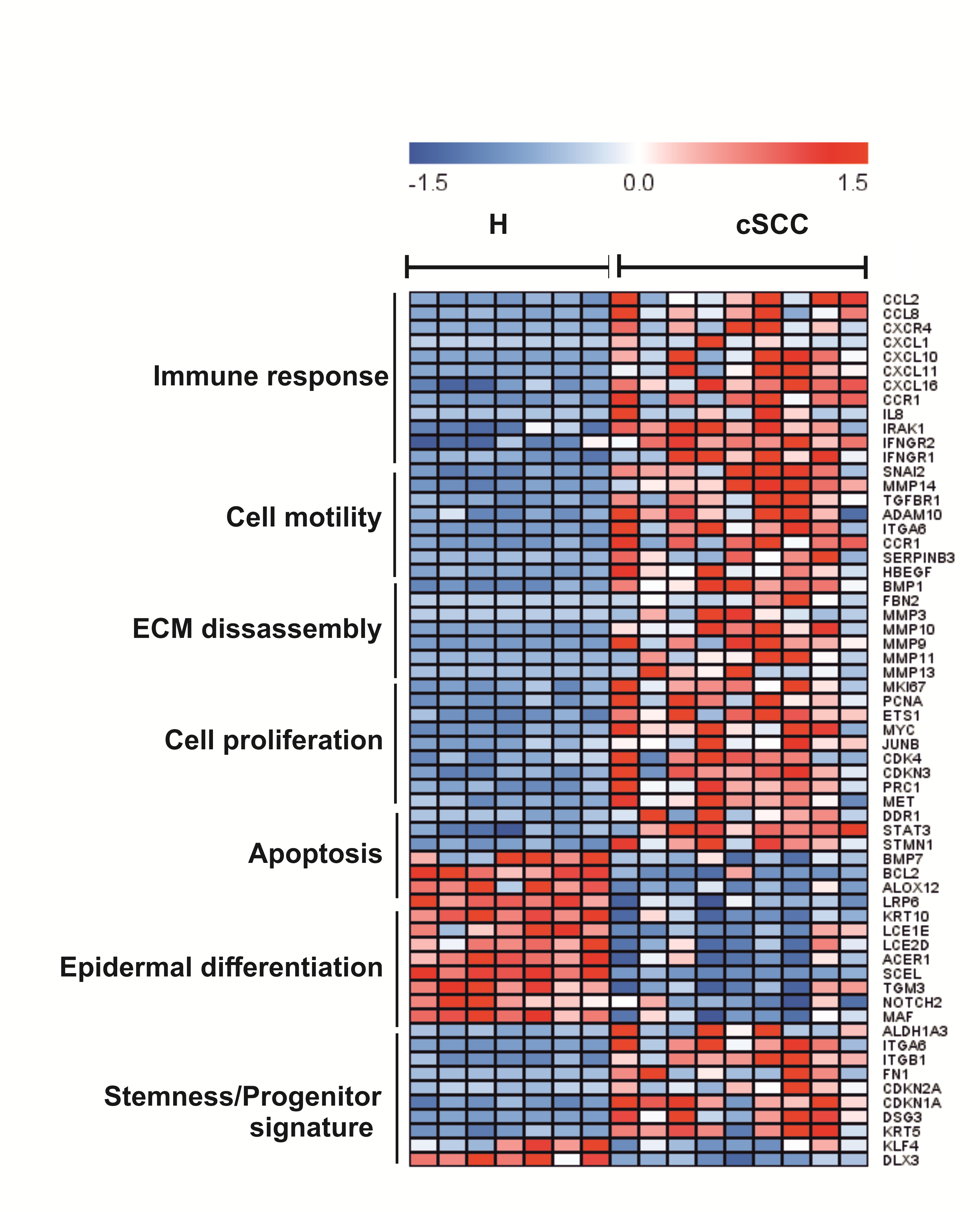
**

**Figure S1**: Heat-map showing the expression pattern of key genes with known function in tumorigenic transformation process in cutaneous squamous cell carcinoma (*P <* 0.05).


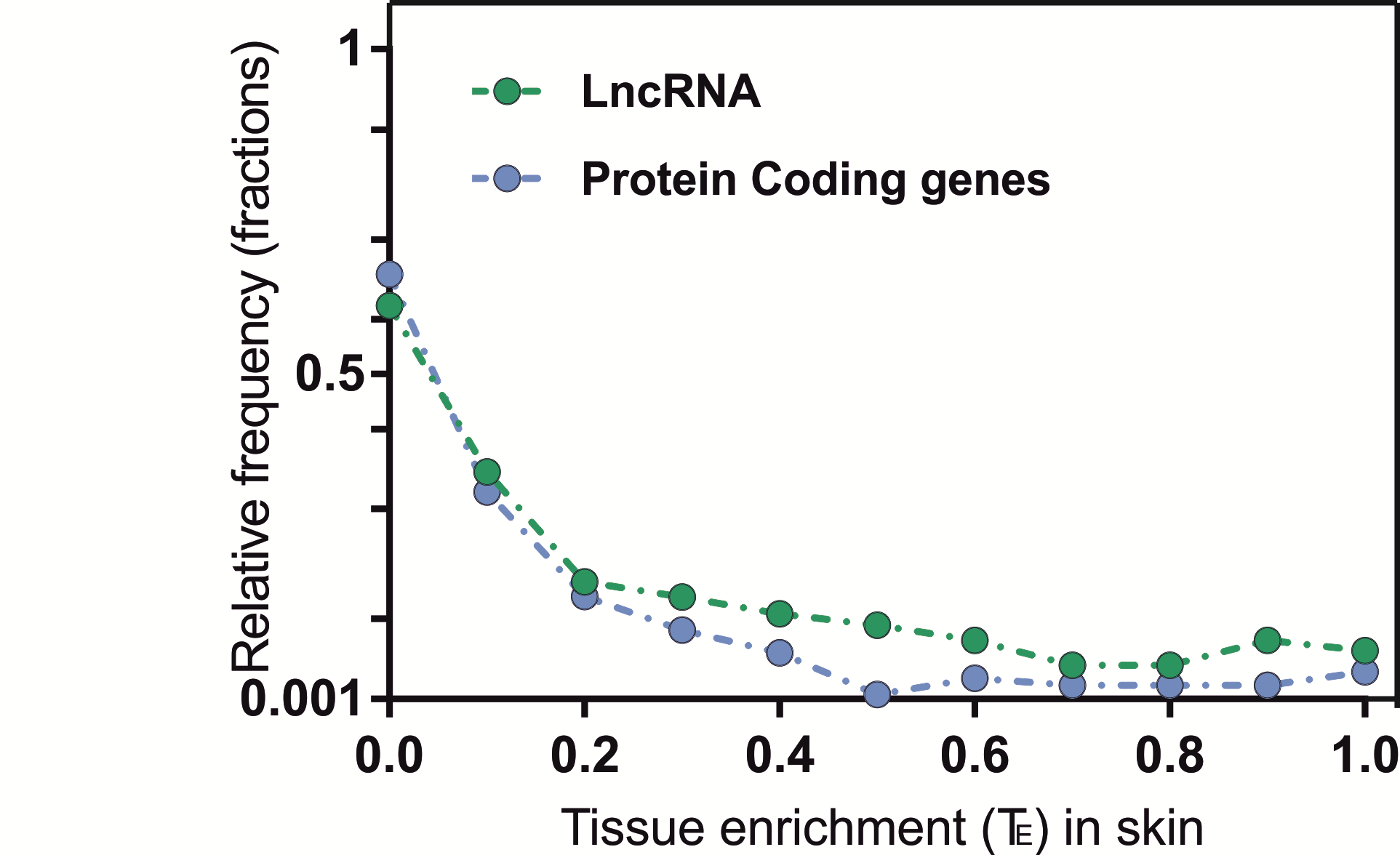


**Figure S2.** Frequency distribution of skin enrichment (T_s_) score for protein coding genes and lncRNAs (0 represents no specificity and 1 represents highest specificity in skin).


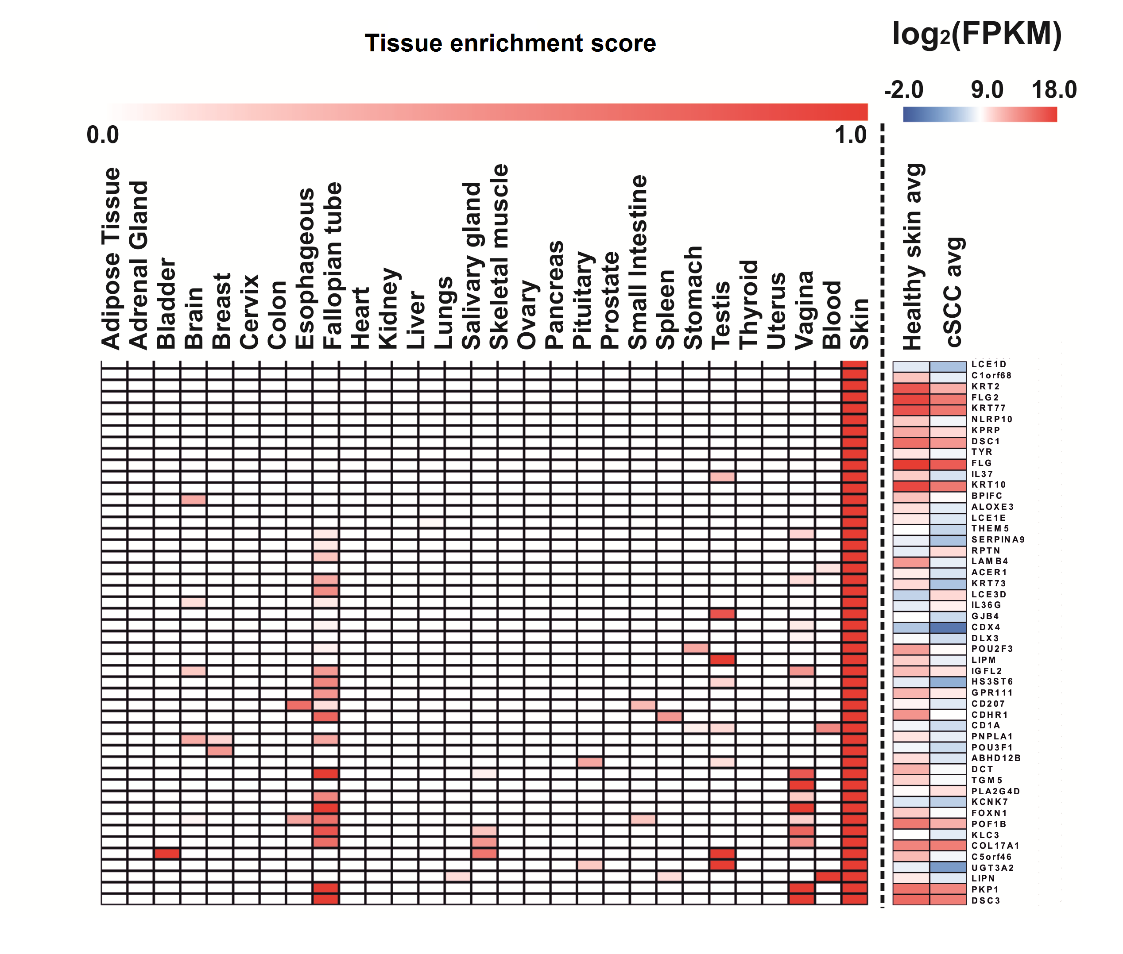


**Figure S3. Tissue specificity analysis of differentially expressed protein coding genes:** Heatmap showing the enrichment (T) of top 50 skin enriched protein coding genes in different tissue types and their expression in healthy skin and cSCC samples.


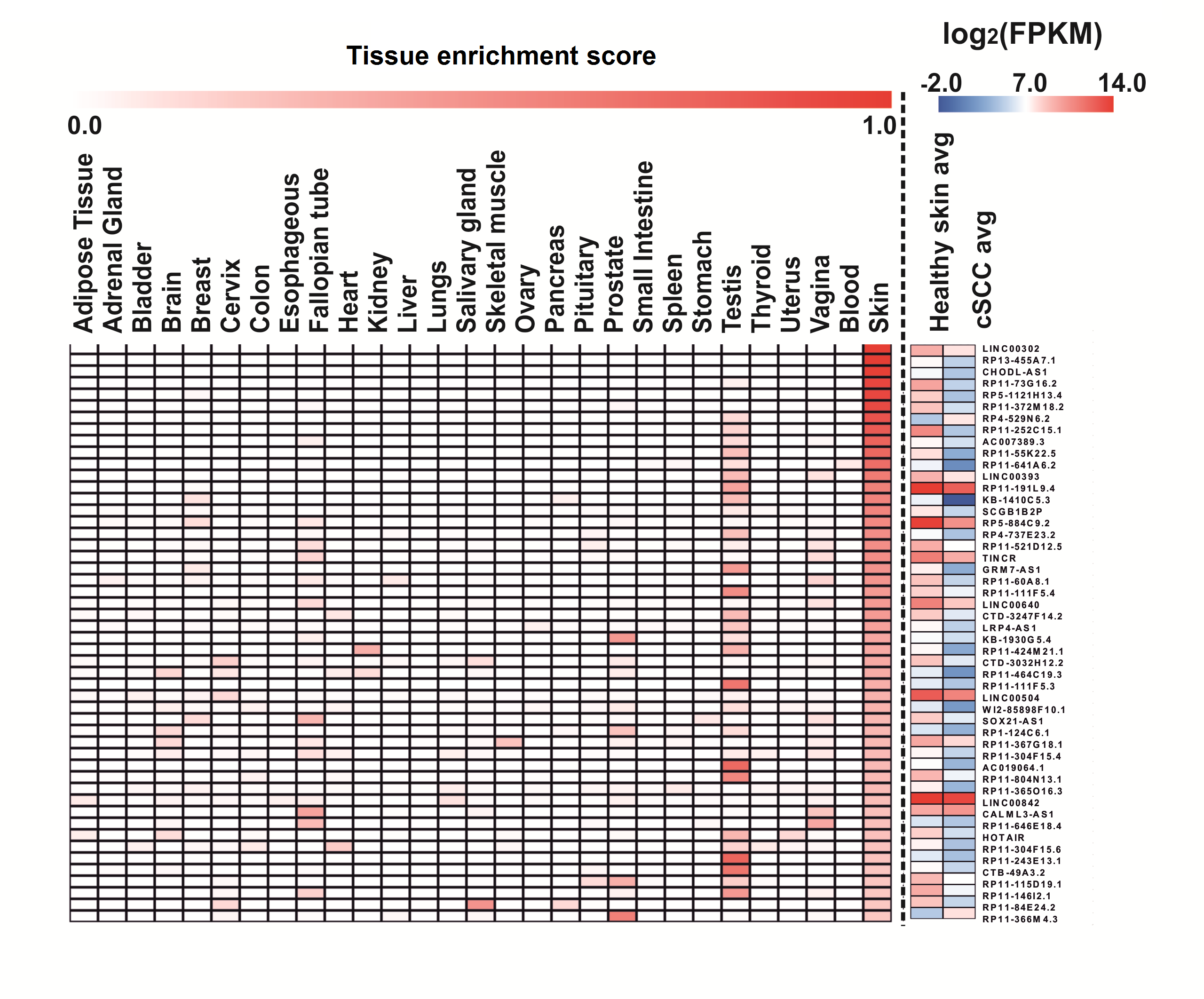


**Figure S4. Tissue specificity analysis of differentially expressed long non coding RNAs:** Heatmap showing the enrichment (T) of top 50 skin enriched lncRNAs in different tissue types and their expression in healthy skin and cSCC samples.


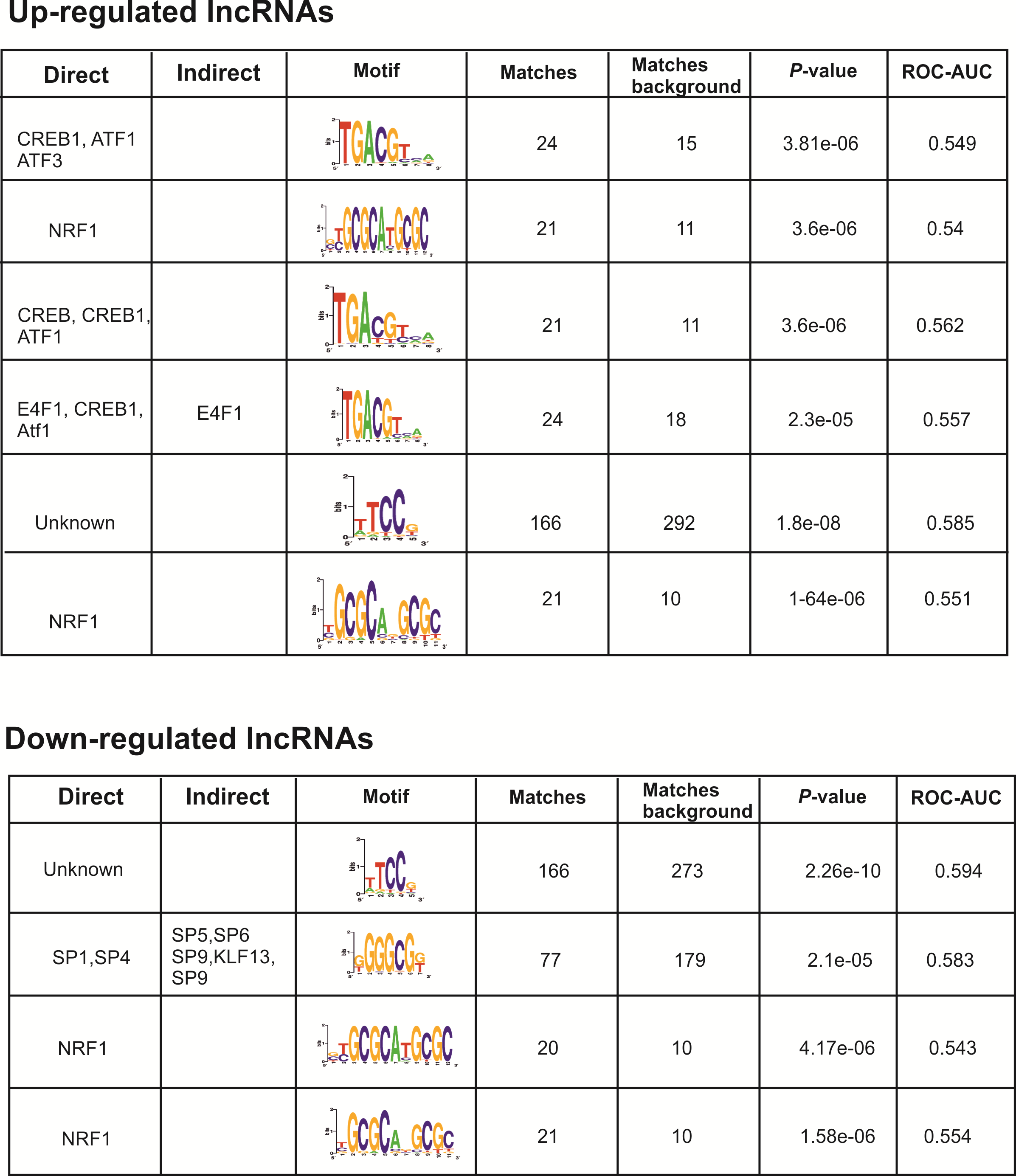


**Figure S5. Motif enrichment analysis of up and downregulated lncRNAs**. The tables show the outcome of a search for known motifs using Gimmeroc from Gimmemotifs. Background: 1000 random 1000bp-sequences from the human genome (hg19) with similar GC content as the DE lncRNA promoters.


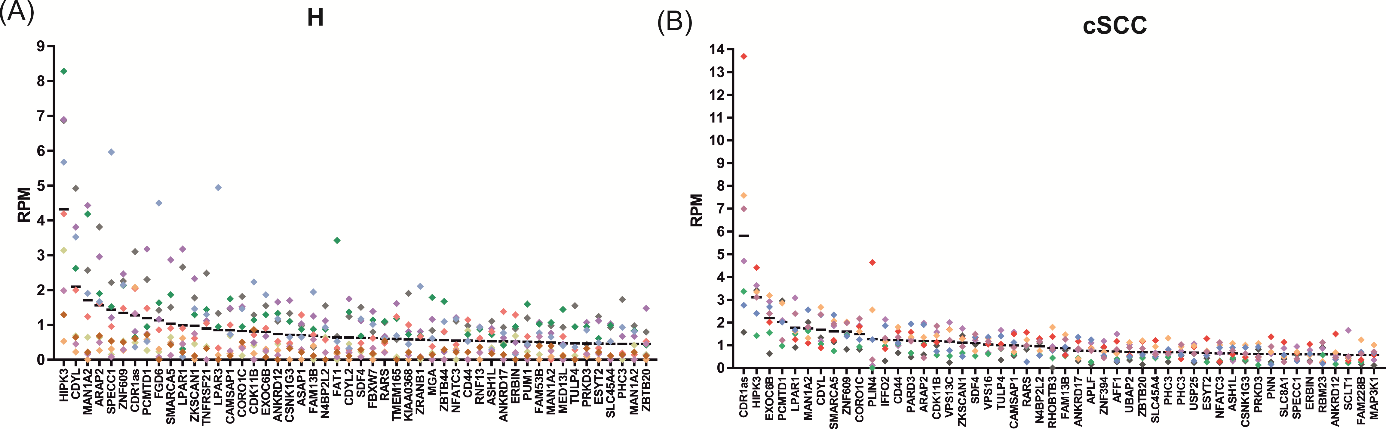


**Figure S6.** **Expression levels of the top 50 most highly expressed circRNAs** in healthy skin **(A)** and cSCC samples **(B)**


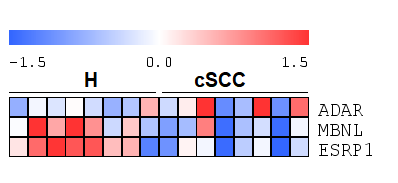


**Figure S7.** Heatmap showing the normalized expression pattern of differentially expressed circRNA regulators (FCH>1.5 and FDR<.05) in healthy skin and cSCC samples

**Figure S8. CircRNA expression changes are largely independent on expression of their linear host genes.** Scatter plot of fold changes in reads per million (RPM) versus fold changes in circular-to-linear (CTL) ratios of the 264 unique high abundance circRNAs with the exception of circRNAs that were not expressed in either of the sample groups. Corresponding linear regression statistics and R-squared values are shown. CDR1as as well as the circRNAs downregulated by more than two fold, which are not present in circBase, are indicated by color.


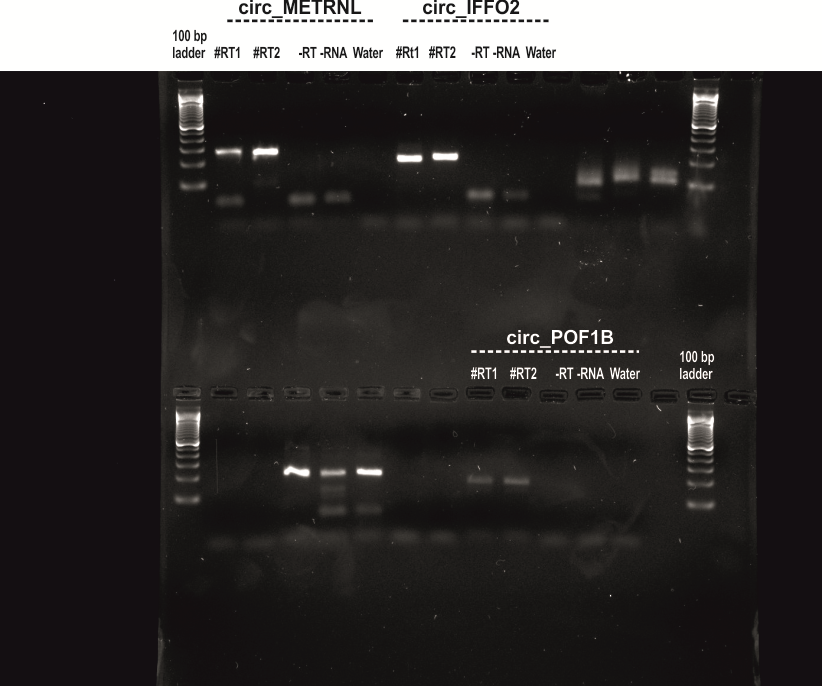


**Figure S9.** Agarose gel image showing the PCR products of novel circRNAs

**REFERENCES**

1. Trapnell, C. *et al.* Differential gene and transcript expression analysis of RNA-seq experiments with TopHat and Cufflinks. *Nat Protoc* **7**, 562–578 (2012).

2. Robinson, M. D., McCarthy, D. J. & Smyth, G. K. edgeR: a Bioconductor package for differential expression analysis of digital gene expression data. *Bioinformatics* **26**, 139–140 (2010).

3. Venø, M. T. *et al.* Spatio-temporal regulation of circular RNA expression during porcine embryonic brain development. *Genome Biology* **16**, (2015).

4. Zhang, X.-O. *et al.* Complementary Sequence-Mediated Exon Circularization. *Cell* **159**, 134–147 (2014).

5. Kristensen, L. S., Okholm, T. L. H., Venø, M. T. & Kjems, J. Circular RNAs are abundantly expressed and upregulated during human epidermal stem cell differentiation. *RNA Biol* **15**, 280–291 (2018).

6. Chen, E. Y. *et al.* Enrichr: interactive and collaborative HTML5 gene list enrichment analysis tool. *BMC Bioinformatics* **14**, 128 (2013).

7. Quinlan, A. R. & Hall, I. M. BEDTools: a flexible suite of utilities for comparing genomic features. *Bioinformatics* **26**, 841–842 (2010).

8. van Heeringen, S. J. & Veenstra, G. J. C. GimmeMotifs: a de novo motif prediction pipeline for ChIP-sequencing experiments. *Bioinformatics* **27**, 270–271 (2011).
